# High Energy Diets-Induced Metabolic and Prediabetic Painful Polyneuropathy in Rats

**DOI:** 10.1371/journal.pone.0057427

**Published:** 2013-02-25

**Authors:** Fang Xie, Han Fu, Jun-Feng Hou, Kai Jiao, Michael Costigan, Jun Chen

**Affiliations:** 1 Institute for Biomedical Sciences of Pain and Institute for Functional Brain Disorders, Tangdu Hospital, The Fourth Military Medical University, Xi'an, P. R. China; 2 Key Laboratory of Brain Stress and Behavior, PLA, Xi'an, PR China; 3 Department of Endocrinology, Tangdu Hospital, The Fourth Military Medical University, Xi'an, P. R. China; 4 F.M. Kirby Neurobiology Center, Children's Hospital Boston, Boston, Massachusetts, United States of America; Torrey Pines Institute for Molecular Studies, United States of America

## Abstract

To establish the role of the metabolic state in the pathogenesis of polyneuropathy, an age- and sex-matched, longitudinal study in rats fed high-fat and high-sucrose diets (HFSD) or high-fat, high-sucrose and high-salt diets (HFSSD) relative to controls was performed. Time courses of body weight, systolic blood pressure, fasting plasma glucose (FPG), insulin, free fatty acids (FFA), homeostasis model assessment-insulin resistance index (HOMA-IR), thermal and mechanical sensitivity and motor coordination were measured in parallel. Finally, large and small myelinated fibers (LMF, SMF) as well as unmyelinated fibers (UMF) in the sciatic nerves and ascending fibers in the spinal dorsal column were quantitatively assessed under electron microscopy. The results showed that early metabolic syndrome (hyperinsulinemia, dyslipidemia, and hypertension) and prediabetic conditions (impaired fasting glucose) could be induced by high energy diet, and these animals later developed painful polyneuropathy characterized by myelin breakdown and LMF loss in both peripheral and central nervous system. In contrast SMF and UMF in the sciatic nerves were changed little, in the same animals. Therefore the phenomenon that high energy diets induce bilateral mechanical, but not thermal, pain hypersensitivity is reflected by severe damage to LMF, but mild damage to SMF and UMF. Moreover, dietary sodium (high-salt) deteriorates the neuropathic pathological process induced by high energy diets, but paradoxically high salt consumption, may reduce, at least temporarily, chronic pain perception in these animals.

## Introduction

International Diabetes Federation Diabetes Atlas estimates that by 2011, 366 million people worldwide have diabetes (accounting for 8.3% of adults) and diabetic patients are expected to increase to 552 million people by 2030 [1]. Global data from systematic analysis of health examination surveys and epidemiological studies with 370 countries and 2.7 million participants across 1980–2008 showed not only an increase in diabetes prevalence but also a rise in fasting plasma glucose (FPG) per decade, since 1980 [2], suggesting a potential increase in prediabetes. Prediabetes is referred to as an intermediate group of individuals whose blood glucose concentrations are higher than normal, but lower than the diabetes threshold [3]–[5]. Prediabetes has also been defined as impaired fasting glucose (IFG) or impaired glucose tolerance (IGT) [3], [6]. It has been generally accepted that prediabetes with IFG or IGT is a high risk factor of developing type 2 diabetes mellitus (T2DM) and cardiovascular diseases [1], [3]–[6] despite some reports arguing that IGT had higher sensitivity for prediction of T2DM [7]–[9]. In China, along with 9.7% diabetes prevalence, the number of prediabetic people has been estimated at 148.2 million adults, accounting for 15.5% of the population [10]. Furthermore, the prevalence of both prediabetes and diabetes is age-dependent with the peak of prediabetes (50–60 years old) being ten years earlier than that for diabetes (60–70 years old) [10], [11], supporting the existence of pre-clinical processes in T2DM patients.

Peripheral neuropathy, with clinical manifestations of pain, sensory, autonomic and even motor dysfunctions, is a major common complication of both diabetes and prediabetes [12]–[18]. Previous reports showed that peripheral neuropathy was present in 66% of patients with type 1 DM (T1DM) and 59% of T2DM [18]. Other reports showed that idiopathic peripheral neuropathy was present in 40–50% people with IGT [15], [19]–[24]. However, an age- and sex-matched, population-based MONICA (Monitoring Trends and Determinants on Cardiovascular Diseases)/KORA (Cooperative Research in the Region of Augsburg) study of subjects aged 25–74 years showed prevalence of polyneuropathy in 7.4% of those with normal glucose tolerance, 11.3% of those with IFG, 13.0% of those with IGT, and 28.0% of the diabetic subjects [25]. More recently, another age- and sex-standardized epidemiological study in the She Ethnic Minority Chinese subjects aged ≥20 to ≤80 years old revealed prevalence of polyneuropathy in 16.1% of those had IFG, 13.1% had IGT, 18.6% had both IFG and IGT, and 28.4% had diabetes [11]. Others have questioned the link between prediabetes and polyneuropathy. Hughes and colleagues compared 50 patients with various types of polyneuropathy with 50 control subjects and found that 32% of patients and 14% of the controls had IGT or hyperglycemia [26]. However, these differences were not significant after adjusting for age and sex [26]. Nonetheless, it was interesting to note that trends within these data still support a tight relationship between hypertriglyceridemia and polyneuropathy [26].

Diabetic peripheral neuropathy (DPN) has been generally classified into two subgroups: (1) Typical DPN is a chronic, symmetrical sensorimotor polyneuropathy that is thought to be caused by long-standing hyperglycemia [13], [17]. (2) Atypical DPN differs from the typical DPN in terms of onset, time course, manifestations, and the underlying mechanisms are unknown [13], [17]. Atypical DPN has been suggested to be associated with IFG or IGT although further evidence is required to define this link formally. Nerve and blood vessel structure have been studied in DPN patients recently with evidence suggesting that large fiber demyelination, axonal degeneration and loss, epineurial arterioles obliteration and epineurial inflammation are observed in this syndrome [18]. The spectrum of neuropathy in patients with IGT has also been studied and the results showed damage to various types of peripheral nerve fibers, accounting for 42.3% small sensory fiber neuropathy, 15.3% large sensory fiber neuropathy and 42.3% sensorimotor neuropathy [16]. To establish the role of metabolic state in the pathogenesis of polyneuropathy, we have designed a longitudinal study in Sprague-Dawley male rats fed two kinds of high energy diets: high-fat and high-sucrose diets (HFSD) and high-fat, high-sucrose and high-salt diets (HFSSD). The rats fed conventional diets (CD) were used as control. In these three groups, time courses of body weight, systolic blood pressure (SBP), fasting plasma glucose (FPG), plasma insulin, free fatty acids (FFA), homeostasis model assessment index (HOMA)-IR, thermal and mechanical sensitivity and motor coordinating performance were measured in a parallel manner in a period of 0–120 days. Following these experiments quantitative histopathology of large and small myelinated fibers and unmyelinated fibers in the sciatic nerves as well as ascending spinal dorsal column fibers were assessed under electron microscopy.

## Materials and Methods

### Animals and treatment

The experiments were performed on three-month-old male Sprague-Dawley albino rats (purchased from Laboratory Animal Center of Fourth Military Medical University, FMMU, Xi'an, P.R. China). The animals were housed in plastic cages with access to food and water *ad libitum* and maintained on a 12 h light/dark cycle at room temperature (22–26°C). Somatic functional evaluations were carried out between 9:00 and 18:30. The rats were acclimated to test boxes for 5 days before the first testing day and for more than 30 min on the following each testing day.

The experimental protocols were approved by Institutional Animal Care and Use Committee of FMMU (Permit number: SCXK2007-007). The present study was performed in accordance with the National Institute of Health Guide for the Care and Use of Laboratory Animals (NIH Publications No. 80-23) revised 1996.

Thirty rats were randomly divided into three groups: one control group fed a conventional diet (CD, n = 10); the two treatment groups were fed HFSD (n = 10) and HFSSD (n = 10), respectively. The experiment lasted from post diet day (PDD) 1 to 120. The composition of the CD, HFSD and HFSSD and the mass or energy proportion of carbohydrate, protein and fat in the three groups are shown in supplementary data (Table S1 and Table S2), respectively. Body weight and foodstuff consumption of each rat per day-and-night were measured regularly. Blood was collected for biochemical measurements and analysis of FPG, FFA and insulin regularly after quantitative measurement of somatic functions (for details see below).

### Measurement of SBP

SBP was measured by a computerized multi-channel physio-recording and analytical system (RM-6280, Chengdu Medical Instrument Factory, P.R.China) every 5 days. Rats were measured five times on a fixation time (8:00–10:00) and averaged values were used as mean SBP.

### Blood sampling and plasma assay

All animals underwent a 12 hours overnight fasting before blood collected. Blood was collected every 5 days until PDD 30 by shearing tail tip between 8:00 and 9:00. Blood collection was practiced again at the end of the experiment. FPG was measured with One Touch blood glucose meter (Lifescan Inc., California, U.S.A.) directly. Plasma FFA was measured according to the protocol described by the manufacturer of FFA kit (Applygen Technologies Inc., Beijing, P.R.China). The fasting plasma insulin concentration was also measured according to the protocol described by the manufacturer of^ 125^I-Insulin Radioimmunoassay Kit (Chemclin Biotech Co., Ltd., Beijing, P.R.China).

### Homeostasis model assessment

HOMA model, which incorporates measures of both FPG and insulin levels, was used as an index of IR and calculated with the following formula: [insulin ( µU/ml)×glucose (nM)÷22.5] as described previously [27].

### Quantitative sensory test

Somatic pain sensitivity was evaluated as an index of somatic sensory functions. Paw withdrawal mechanical threshold (PWMT) was measured using ascending graded individual von Frey monofilaments with bending forces of 3.5, 4.5, 5.5, 7.8, 11, 15, 20, 25, 30, 40, 50, 60 g as reported previously [28]. The rat was placed on a metal mesh floor covered with the same plastic chamber and von Frey filaments were applied from underneath the metal mesh floor to the testing site of the bilateral hind paws. A von Frey filament was applied ten times (once every several seconds) to each testing area. The bending force value of the von Frey filament that caused a 50% occurrence of paw withdrawal was expressed as the PWMT. The stimulus was stopped if the threshold exceeded 60 g (cutoff value). Paw withdrawal thermal latency (PWTL) was measured by using a RTY-3 radiant heat stimulator (Xi'an Bobang Technologies of Chemical Industry Co. Ltd., P.R.China) [28]. The radiant heat source was a high intensity halogen lamp bulb (150 W) positioned under the glass floor directly beneath target area on the hind paw. Five stimuli were repeated for each site and the latter three values were averaged as the mean PWTL. The inter-stimulus interval was more than 10 min. The thermal latency was defined as the duration from the onset of heat stimulus to the occurrence of hind paw withdrawal reflex. The stimulus was stopped if the latency exceeded 30 s so as to avoid excessive tissue injury. Percentage change in PWTL (s) or PWMT (g) to heat stimulus or von Frey filament stimulus was obtained by: % change = PWTL or PWMT - baseline)/baseline ×100%.

### Assessment of motor coordinating performance

Motor coordinating performance was tested using a Rota-rod treadmill (Ugo, Ltd., Italy). The rats were tested simultaneously on the apparatus with a rod-rotating speed of 6 rpm. The accelerating speed of the Rota-rod was set to increase from 6 rpm to 30 rpm within 2 min. The animals were placed on the treadmill and the timers were started with acceleration and automatically stopped when the animal fell off, with a maximal cutoff time of 300 s.

### Electron microscopic examination of somatic nerve fibers

At the end of the whole experiment, three rats per group were infused with 2.5% glutaraldehyde and 4% paraformaldehyde in 0.1 M phosphate buffer (pH = 7.4) after anesthetization with sodium pentobarbital (80 mg/Kg; Sigma, Ltd., U.S.A). Then the sciatic nerves and spinal dorsal column were collected and postfixed by a fixative of 3% glutaraldehyde in 0.1 M phosphate buffer (pH = 7.4), overnight at 4 °C. Transverse sections (1 mm) of the spinal cord were obtained by vibrating microtome DTK-1000 (Dosaka, Ltd., Japan). The dorsal column were dissected out and cut into small pieces of similar dimensions, followed by osmification in 1% OsO_4_ in 0.1 M sodium cacodylate buffer for 2 hours at room temperature, dehydration in an ascending acetone series. The small-cutting-blocks of sciatic nerve were prepared in the same osmification and dehydration procedure. The osmicated tissue blocks were further embedded in Epon-812 (Serva, Ltd., Germany) and trimmed carefully under the light microscope. Ultrathin sections (50–70 nm) were cut perpendicularly to the axis of nerve fibers with a diamond knife on ultramicrotome LKB-11800 (LKB Ltd., Sweden) and collected by copper grids (300 meshes). The ultrathin sections stained with uranyl acetate and lead citrate were observed and microphotographs were taken under an electron microscope (EM, JEM-2000EX, JEOL Ltd., Japan).

### Histopathological evaluation

When electron photomicrographs were taken, the areas of each axon and fiber profile were measured using Image Pro Plus 6.0, and the area of each fiber myelin profile was obtained based upon the formula: area of myelin profile  =  area of fiber profile–area of axon profile. The profile ratio was referred to as a ratio between the area of an axon profile and the area of a fiber profile according to the formula of the conventional G-ratio. However, because G-ratio is obtained by a ratio between circumference of an axon and circumference of a fiber (2πR of an axon/2πR of a fiber = radius of an axon/radius of a fiber) and the radius of the damaged fibers would produce great bias due to irregularity of the fiber shapes, it was not adopted in the current study.

The damaged fibers were also classified into four grades according to the intensity and extensity of destruction of myelinated and UMF axons for both the SN and the SDC (Fig. S1). The pathologically-classified grade I (pI) is referred to as a slight pathological change, including myelin lamina rarefaction, decompaction, focal demyelination or vacuolization but axon is normal on the whole. pII includes myelin lamina reticulating, focal demyelination or vacuolization and slight changes occurring in axon, such as lipofuscin deposition, glycogen granules, dense microfilament and microtubule. pIII includes wide focal demyelination and big vacuolization encroaching on axon, compressed axon and other scenes seen in grade II. pIV is a severe pathological change: both myelin sheath and axon were destructed or lost, including myelin breakdown and big vacuolization, axonal degeneration and even axonal loss. In each animal, more than 150 individual LMF (Φ6∼20 µm, including Aα, Aβ fibers), SMF (Φ1.6∼5 µm, equals to Aδ fibers) and UMF (Φ0.2∼1.5 µm, C fibers) in the SN and more than 300 individual myelinated fibers in the SDC were measured and analyzed. The data used in the current study were from three rats per group.

### Statistical analysis

Shapiro-Wilk normality test was first used to determine which data are normally or non-normally distributed. Normally distributed data were shown as mean±SEM, while non-normally distributed data were shown as median with maximum and minimum. Parametric one-way ANOVA followed by Fisher's PLSD post hoc analysis was used for comparisons of the normally distributed data (body weight, SBP, biochemistry parameters, and somatic sensorimotor behaviors) between CD and HFSD and HFSSD groups. Non-parametric Kruskal-Wallis H test and Mann-Whitney U test were used for comparisons of non-normally distributed data (areas of myelin profiles, profile ratios, and proportions of the damaged nerve fibers) between CD and HFSD and HFSSD groups. Value p<0.05 was considered to be significantly different.

## Results

### Induction of metabolic syndrome and prediabetes in rats by diet

#### Foodstuff consumption and body weight

**Figure 1:**
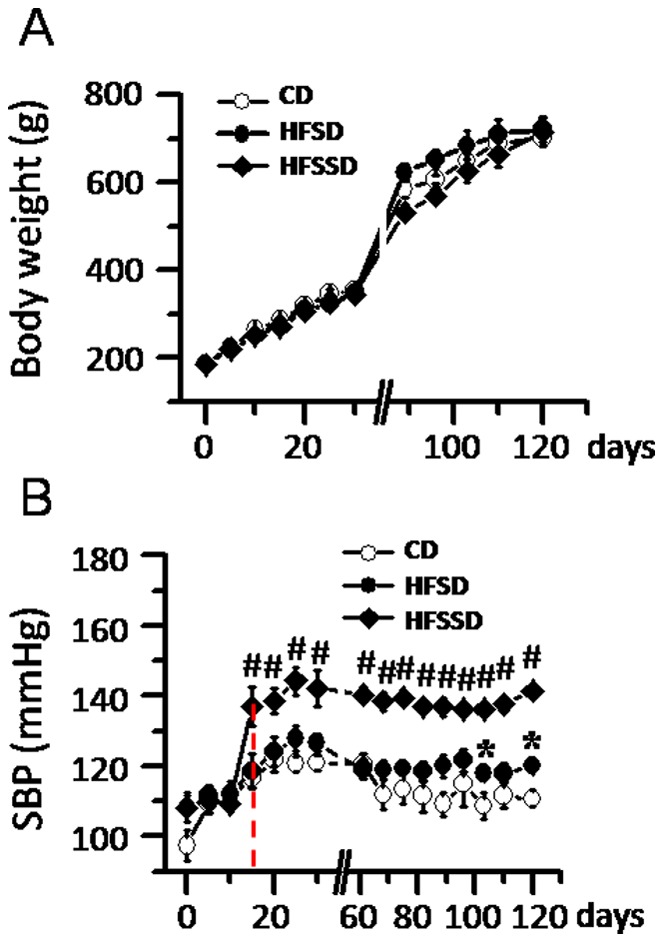
Time courses of the effects of CD, HFSD and HFSSD diets on body weight (A) and systolic blood pressure (SBP) (B). CD, conventional diet; HFSD, high-fat and high-sucrose diets; HFSSD, high-fat, high-sucrose and high-salt diets. Vertical dashed line indicates initial significant changes in SBP. Ten rats were used in each diet group for this statistical analysis across post diet days 0–120. ^*^ *P*<0.05, HFSD vs. CD; # *P*<0.05, HFSSD vs. CD. Error bars: ± SEM.

The average foodstuff consumption per rat per day was measured across the whole time course from PDD 0 to 120. Rats fed CD diet consumed more foodstuff (35.3±0.3 g/day, n≥6) than either of the rats fed HFSD (29.2±0.2 g/day, n≥5) or HFSSD (28.7±0.2 g/day, n≥5) (*P*<0.05). Nonetheless, for the calorie intake, there was no significant difference among groups of rats fed CD, HFSD and HFSSD. For body weight shown in Fig. 1A, there was no significant difference among the three diet groups from the beginning till the end of the experiment, although the body weights of the three groups of rats were increased significantly from PDD 0 (CD, 188.2±3.0 g, n = 10; HFSD, 187.5±2.5 g, n = 10; HFSSD, 185.6±2.4 g, n = 10) to PDD 120 (CD, 701.7±9.5 g, n = 6; HFSD, 723.3±25.7 g, n = 5; HFSSD, 715.0±30.5 g, n = 5). Four rats in each group were sacrificed for intravenous glucose tolerance test on PDD 30. Besides, one animal died before the experimental termination within HFSD and HFSSD groups respectively.

#### Changes in systolic blood pressure

SBP of the three groups were measured every 5 days from the beginning till the end of the experimental observation. Rats fed CD showed a slight increase in SBP from PDD 5 to 120 within normal range (Fig. 1B). Comparing the averaged values of SBP, there was no significant difference between that of rats fed CD and HFSD (97.5 to 121.7 mmHg, n≥6 vs. 108∼127.8 mmHg, n≥5, *P*>0.05), although significant increases were observed from PDD 105 to 120. However, rats fed HFSSD showed significant increases in SBP values from PDD 15 until the end of the experiment (PDD 0 vs. PDD 120: 104.0∼111.7 mmHg, n = 10 vs. 136.1∼144.2 mmHg, n = 5) (Fig. 1B).

#### Changes in fasting plasma glucose

**Figure 2:**
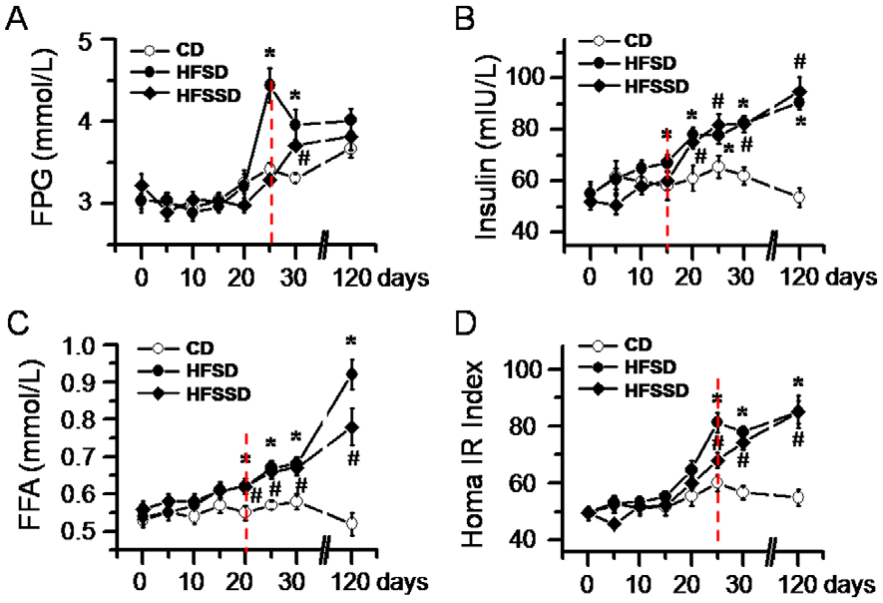
Time courses of the effects of CD, HFSD and HFSSD diets on blood biochemistry. The fasting plasma glucose (FPG, A), the plasma insulin (B), the free fatty acids (FFA, C) and the homeostasis model assessment insulin resistance (Homa-IR) index (D) are shown. At least five rats were used in each diet group for this statistical analysis across post diet days 0–120. ^*^ *P*<0.05, HFSD vs. CD; # *P*<0.05, HFSSD vs. CD. Vertical dashed lines indicate initial significant changes in blood chemistry caused by the diets. For abbreviations see Fig.1. Error bars: ± SEM.

The FPG of rats among the three groups was measured every 5 days from PDD 0 to 30, and PDD120 before termination of the experiment. Rats fed CD showed no change in FPG from PDD 0 to 20 followed by a slight elevation from PDD 25 to 120 (Fig. 2A). FPG values in rats fed HFSD were not changed from PDD 0 to 20 (Fig 2A). However, the FPG level was significantly increased from PDD 25 and maintained relatively higher level until PDD 120 (Fig. 2A). In rats fed HFSSD, the level of FPG became significantly elevated on PDD 30 compared with the CD group although there was no significant difference between them again on PDD 120 (Fig. 2A). Nonetheless, the FPG values in all of the three groups were maintained below the level of hyperglycemia.

#### Changes in plasma insulin and free fatty acids

In parallel with FPG, plasma insulin and FFA were also measured every 5 days from PDD 0 to 30, and again on PDD 120. In comparison with the rats fed CD, the plasma level of insulin was not significantly changed before PDD 15 in rats fed HFSD and before PDD 20 in rats fed HFSSD (Fig. 2B). However, the plasma levels of insulin were dramatically increased for both high energy diet groups post PDD 25. The increase in plasma insulin levels was time-dependent in both the high energy diet groups of rats and the hyperinsulinemia was maintained linearly until the termination of the experiment (Fig. 2B). There was no significant change in plasma insulin levels during the whole time course in rats fed CD (Fig. 2B).

The plasma levels of FFA were also unchanged until PDD 20 in rats fed both HFSD and HFSSD compared to the CD group (Fig. 2C). However, plasma FFA was significantly increased after PDD 20 for both the high energy diet groups (Fig. 2C). Like insulin, there was no significant change in FFA levels during the whole time course in rats fed CD (Fig. 2C).

#### HOMA-IR index

HOMA-IR index was calculated based upon the concentrations of FPG and plasma insulin and this measure is thought to represent insulin sensitivity (Fig. 2D, for details see Methods). As shown in Fig. 2D, HOMA IR index was not changed from PDD 0 to 120 in rats fed CD, however, the index became significantly increased in both rats fed HFSD and HFSSD after PDD 25, implicating existence of IR in both of the two diet groups. The increased HOMA-IR index was also time-dependent and remained significantly elevated until the termination of the experiment on PDD 120.

### Long-term evaluation of somatic sensorimotor functions

#### Pain sensitivity

**Figure 3:**
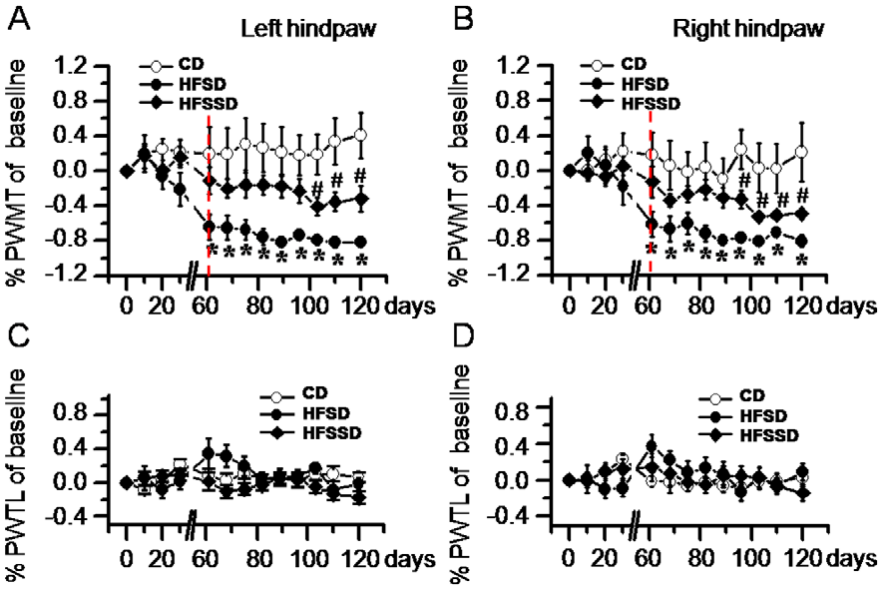
Time courses of the effects of CD, HFSD and HFSSD diets on the somatic sensory functions measured in conscious rats. The somatic sensory function was shown by changes in mechanical pain sensitivity (A-B) and thermal pain sensitivity (C-D) in bilateral hind paws (A and C for left hindpaw and B and D for right hindpaw). PWMT, paw withdrawal mechanical threshold; PWTL, paw withdrawal thermal latency. Five to six rats were used in each diet group for this statistical analysis across post diet days 0–120. ^*^ *P*<0.05, HFSD vs. CD; ^#^ *P*<0.05, HFSSD vs. CD. Vertical dashed lines in A and B indicate initial significant changes in PWMT caused by the diets. For abbreviations see Fig.1. Error bars: ± SEM.

A parallel evaluation of somatic pain sensitivity was performed in each group of rats and bilateral PWMT and PWTL were measured over the whole time course. In rats fed CD, neither PWMT nor PWTL was significantly altered from PDD 0 to 120 (Fig. 3). In rats fed HFSD, however, bilateral PWMT became significantly reduced by 62–82% from PDD 60 and this increased mechanical pain sensitivity continued until the end of the experiment (Fig. 3A-B). In rats fed HFSSD, the PWMT of bilateral hind paws was also decreased, although more gradually than in the HFSD animals, with significantly distinct mechanical hypersensitivity only observed from PDD 95 (Fig. 3A-B). Unlike PWMT, PWTL of bilateral hind paws was not significantly altered across the whole time course in all three diet groups (Fig. 3C-D).

#### Motor coordinating performance

**Figure 4:**
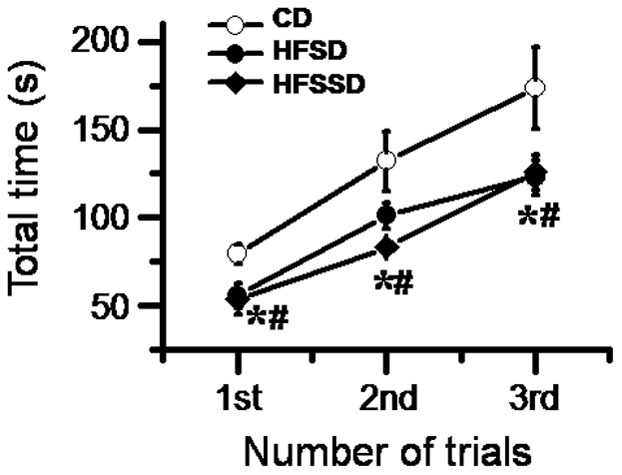
The effects of CD, HFSD and HFSSD diets on the somatic motor functions measured in conscious rats. The somatic motor function was shown by motor coordinating performance of rats on a treadmill on post diet days 120. Five to six rats were used in each diet group for this statistical analysis. ^*^ *P*<0.05, HFSD vs. CD; # *P*<0.05, HFSSD vs. CD. For abbreviations see Fig.1. Error bars: ± SEM.

To exclude motor modulation of sensory input, motor coordinating performance was measured on PDD 120 after sensory evaluation (Fig. 4). It was shown that rats in all three diet groups displayed a trial-dependent increase in the time spent on the treadmill, implicating intact cognitive and behavioral task adaptation. However, rats fed HFSD and HFSSD showed impaired motor coordinating performance compared with those fed CD evidenced by the trial-performance curve for both HFSD and HFSSD groups being shifted rightward relative to the CD group (Fig. 4).

### Evaluation of pathological changes in somatic nervous system

#### Pathological changes in sciatic nerve

**Figure 5:**
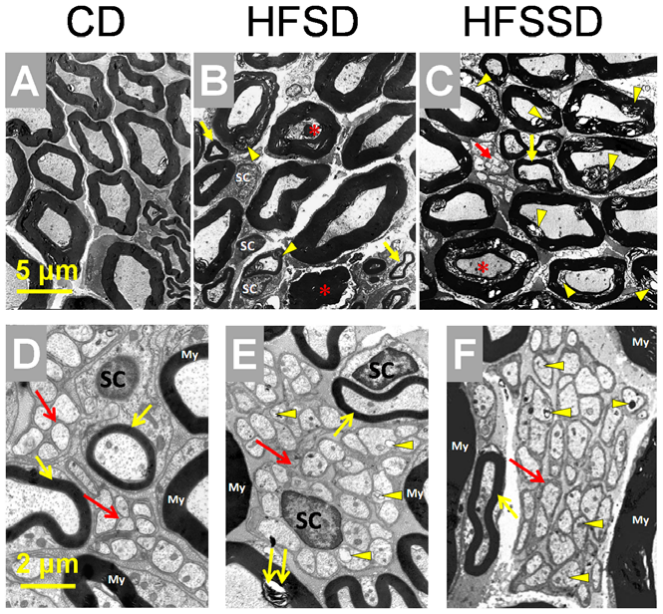
The effects of CD, HFSD and HFSSD diets on the myelinated and unmyelinated fiber structures in the sciatic nerves. Electron microscopic photomicrographs show the cross-section of the sciatic nerve fibers in the CD (A and D), HFSD (B and E) and HFSSD (C and F) groups. Dramatic pathological changes characterized by myelin breakdown or disruption and axon degeneration are mainly seen in large myelinated fibers (LMF) of rats fed HFSD (B) and HFSSD (C) when comparing with CD (A). The diets-induced LMF myelin changes are often seen as myelin lamina rarefaction, focal demyelination and vacuolization (yellow arrowheads in B-C). Axon degeneration of LMFs is characterized by abnormal high electron density and axonal plasmic shrinkage (asterisks in B-C). Ultrastructures of small myelinated fibers (SMF) and unmyelinated C fibers (UMF) in three groups are also shown (D-F). The axolemma and the Schwann cell covering are well maintained in all UMFs of rats fed CD (see red arrows in D), however, the axolemma and the Schwann cell membrane are thickened and perturbed shown as high electron density in both HFSD and HFSSD (red arrows in E-F). In addition, enlarged mitochondria and lipofuscin depositions are also seen in UMF axons of high energy/salt-treated rats (see yellow arrowheads in E-F) but not in control rats (D). The ultrastructures of SMFs in HFSD and HFSSD are well preserved when compared with CD (see yellow arrows in B-F), however, broken SMF can also be seen in the diet rats (double yellow arrows in E). My, myelin sheath; SC, the nucleus of Schwann cells; other abbreviations see Fig.1. Scale bar for A-C: 5 µm, scale bar for D-F: 2 µm.

**Figure 6:**
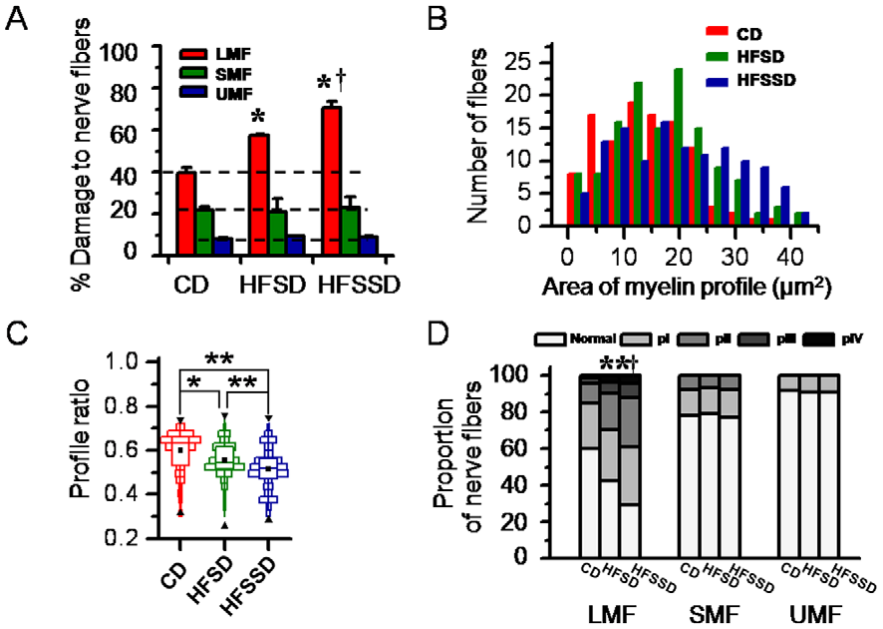
Quantitative analysis of structural changes in LMF, SMF and UMF in the sciatic nerves of rats fed CD, HFSD and HFSSD, respectively. (A) Percent damage to LMF, SMF and UMF. (B) Distributional histograms of profile areas of myelinated fibers. (C) Boxplots show changes in profile ratios (changes in myelin profile) obtained by dividing area of axon profiles with area of fiber profiles of the myelinated fibers. (D) Proportion of nerve fibers with different pathologically-classified grades (grades pI-IV, for details see Fig. S1). Horizontal lines in A indicate level of % damage to nerve fibers in CD. Upper reverse black triangles, lower upright black triangles, horizontal lines across each box and black squares in each box represent maximum, minimum, median and mean of profile ratios in C. For abbreviations see Figs.1 and 5. ^*^ and ^†^ *p*<0.05, ^**^ *p*<0.01. Error bars in A:±SEM.

The ultrastructure of bilateral sciatic nerves of rats fed CD (n = 3), HFSD (n = 3) and HFSSD (n = 3) were examined after PDD 120 under EM. As shown in Fig. 5A-C, there were two major distinct morphological changes in the sciatic nerves of both altered diet groups compared to the CD group: (1) dramatic loss of inter-fiber matrix and consequently enlarged inter-fiber space; (2) dramatic disruption or breakdown of myelin sheath of the LMF. Under EM, the damaged LMF was frequently seen and featured as myelin breakdown, including lamina decompaction, focal demyelination, vacuolization, myelin sheath infolding, axonal inclusion of exfoliate myelin, allogenic myelin inclusions (Fig. 5B-C and Fig. S1A). Among the damaged LMF, axonal degeneration or loss was also frequently observed along with damaged mitochondria, lipofuscin deposition, glycogen granules, vacuolar degeneration, and even axonal structural disappearance (Fig. 5B-C and Fig. S1A). Moreover, the high sodium diet (HFSSD) exaggerated these pathological changes when compared to the HFSD group (Fig. 5B-C). Quantitatively, the damaged LMF were 39.8% in the CD, 57.4% in the HFSD and 71.0% in the HFSSD (Fig. 6A).

However, compared with the severe disruptions present in the LMF, only mild pathological alterations were found in the SMF and the UMF of the sciatic nerves in rats fed HFSD and HFSSD compared to control (Fig. 5D-F). Morphologically, SMF remained mostly intact in rats fed CD, HFSD or HFSSD (see yellow arrows in Fig. 5B-F). In agreement with these images, quantitative analysis (Fig. 6A) displayed no significant difference in the amount of damaged SMF across the three diet groups (21.9±1.7%, 20.8±6.7%, 23.0±5.3% for CD, HFSD and HFSSD, respectively, n≥6, p>0.05). It was shown however, that the ultrastructure of the UMF was slightly altered in rats fed both the HFSD (Fig. 5E) and the HFSSD (Fig. 5F) compared with the CD (Fig.5D). Comparing with the CD (see red arrows in Fig. 5D), the thickening (increased electron density) in axolemma of UMF and the Schwann cell covering was common in rats fed both the HFSD and the HFSSD (see red arrows in Fig.5E-F). The lipofuscin deposition and enlarged mitochondria could also be found in these UMF axons occasionally (see yellow arrowheads in Fig. 5E-F). The altered ultrastructure in the UMF of HFSSD group was more severe than the HFSD (Fig.5E-F). Although slight damage was evident in the two altered diet groups relative to CD within the photomicrographs, when quantified global levels of damaged UMF did not differ significantly across the three groups (8.1±0.5%, 9.2±0.2%, 9.0±0.5% for CD, HFSD and HFSSD, respectively, n≥6, p>0.05, see Fig. 6A).

Quantitative analysis of structural changes in myelinated fibers in the sciatic nerves of rats fed CD, HFSD and HFSSD are shown in Figure 6. As discussed above, pronounced levels of damage are present in the LMF of both high energy diets relative to controls, with the additional salt diet showing the highest levels of injury (Fig 6A). In contrast, no significant differences were seen in either the SMF or the UMF in any of the diet groups, although subtle trends toward more damage in the high energy diets relative to controls were witnessed. A tendency of increased area of myelin present within each fiber profile counted is evident in the HFSD and the HFSSD groups when comparing to the CD group (Fig. 6B). Moreover, the profile ratios (changes in myelin profile) were significantly decreased in the sciatic nerves of rats fed HFSD and HFSSD when comparing with the CD (Fig. 6C). Or in other terms, more abnormal myelin signal was evident within the nerve fibers of the high energy diets at the end of the experiment relative to the beginning when compared with the control diets. These results are consistent with the pathological changes caused by myelin breakdown or loosening and ballooning within the myelin sheath.

Under EM, the damaged fibers were classified into four grades according to the intensity and extensity of destruction of myelin sheath and axons within the sciatic nerve (Fig. S1A). The proportion of the normal and the pathologically-classified (pI-IV) nerve fibers in all of the three groups of rats were shown in Fig. 6D. Based upon this proportional analysis, pathological grades III-IV were mainly seen in the LMF of rats fed HFSD and HFSSD and rarely observed in rats fed CD. Furthermore, rats fed the HFSSD showed more severe myelin breakdown and axonal degeneration than those fed the HFSD. In contrast, nearly 80% of the SMF and more than 90% of the UMF in the peripheral sciatic nerves were intact in either of the three diet groups, suggesting a relative insensitivity of these nerve fibers to the high energy diets tested.

#### Pathological changes in spinal dorsal column

**Figure 7:**
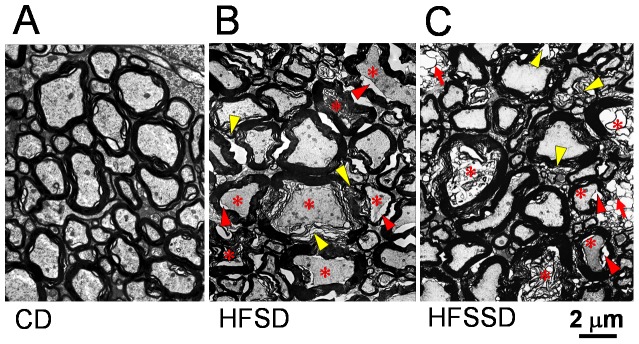
The effects of CD, HFSD and HFSSD diets on the myelinated fiber structures in the spinal dorsal column (DC). Electron microscopic photomicrographs show the cross-section of the DC fibers in the CD (A), HFSD (B) and HFSSD (C) groups. Dramatic pathological changes characterized by myelin breakdown or disruption (yellow arrowheads), split between axon and myelin sheath (red arrowheads) and axon degeneration (asterisks) are mainly seen in large myelinated fibers of rats fed HFSD (B) and HFSSD (C) when comparing with CD (A). Beyond the pathological changes observed in the sciatic nerves (see legend in Fig.5), both axon hypertrophy and atrophy are distinct with the inter-fiber metrix being poorly preserved in high energy/salt rats. Cavitation or balloon-like structures (red arrows) can also be seen in HFSSD group. For abbreviations see Fig.1. Scale bar: 2 µm.

The spinal dorsal column is mainly comprised of central axonal fibers of the primary sensory neurons that innervate peripheral sensory organs perceiving cutaneous touch, pressure and vibration or deep proprioception. The ultrastructure of spinal dorsal column fibers in rats fed CD (n = 3), HFSD (n = 3) and HFSSD (n = 3) were examined as well after PDD 120 under EM. As shown in Fig. 7B-C and Fig. S1B, distinct loss of inter-fiber matrix and dramatic disruption or breakdown of myelin sheath in the myelinated fibers were also evident in rats fed HFSD and HFSSD, when compared with CD (Fig. 7A). Lamina rarefaction, focal demyelination, vacuolization, and even myelin sheath breakdown was frequently seen in the spinal dorsal column of rats fed HFSD and HFSSD (Fig. 7B-C and Fig. S1B). Axon atrophy, axon edema and hypertrophy, axonal degeneration and loss were also frequently observed in the two diet groups (Fig. 7B-C and Fig. S1B). The damaged myelinated fibers also contained lipofuscin deposition, glycogen granules and vacuolar degeneration (Fig. 7B-C and Fig. S1B). Similar to the sciatic nerves, pathological alterations of the inter-fiber matrix, such as the loss of integrity, the disintegrations and the edema of extracellular matrix were also common in the spinal dorsal column of rats fed HFSD and HFSSD (Fig. 7B-C and Fig. S1B).

**Figure 8:**
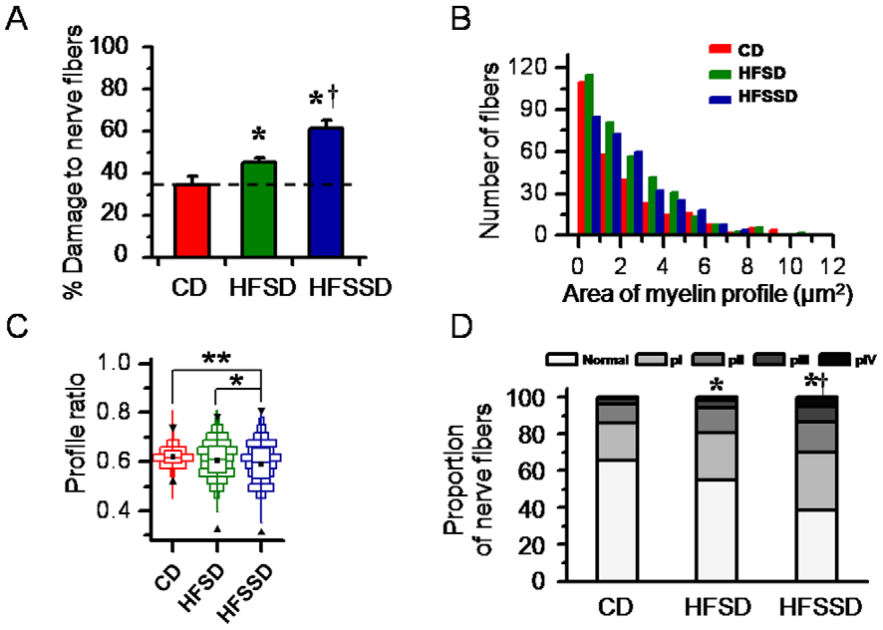
Quantitative analysis of structural changes in myelinated fibers in the spinal dorsal column of rats fed CD, HFSD and HFSSD, respectively. (A) Percent damage to myelinated fibers. (B) Distributional histograms of profile areas of myelinated fibers. (C) Boxplots show changes in profile ratios of the myelinated fibers. (D) Proportion of nerve fibers with different pathologically-classified grades (grades pI-IV, for details see Fig. S1). Horizontal line in (A) indicate level of % damage to nerve fibers in CD. Upper reverse black triangles, lower upright black triangles, horizontal lines across each box and black squares in each box represent maximum, minimum, median and mean of profile ratios in (C). For abbreviations see Figs.1 and 5. ^*^ and ^†^ *p*<0.05, ^**^ *p*<0.01. Error bars in A:±SEM.

As quantified in Fig. 8A, the total amount of damaged myelinated fibers in the spinal dorsal column was significantly increased in the diet groups compared with control (HFSD or HFSSD vs. CD: 45.3±1.9% or 61.2±4.2% vs. 34.4±4.5%, n≥6, p<0.05). A distributional histogram of myelin area per axon profile showed a tendency for increased area of myelin presence in the HFSD and the HFSSD groups when comparing to the CD group (Fig. 8B). The profile ratios were also significantly decreased in the spinal dorsal column of rats fed HFSD and HFSSD when comparing with the CD (Fig. 8C). Suggesting as in the PNS, that more abnormal myelin signal was evident within the nerve fibers of the high energy diets at the end of the experiment relative to the beginning, when compared with the control diets.

Graded quantification of the amount of myelin damage in the spinal dorsal column (Fig. S1B) showed CNS results (Fig. 8D) consistent with those present in the PNS (Fig. 6D); namely, that pathological damage was clearly evident in both high energy diets, but was worst in the high salt group.

## Discussion

### High energy/salt diets-induced metabolic syndrome and prediabetes

During this longitudinal study of rats fed two kinds of high energy (HFSD and HFSSD), versus control diets *ad libitum* we were surprised to note no overall weight gain in the high energy groups relative to controls. We do note however, that the rats opted to consume significantly less of the high energy diets relative to the control diet and therefore this result is consistent with the overall calorific consumption across each group. We note also that these studies were only continued for a relatively short period (4 months) and that if we had extended the time course further we may well have seen significant changes in body weight developing between the groups [29]. In contrast, the results showed a distinct and dramatic increase in the levels of SBP, insulin, FFA and HOMA-IR index attributable to HFSD or HFSSD when compared with those values in rats fed CD suggesting that these metabolic changes, but not obesity, are critical factors in the early processes that lead to pre-diabetes. It will be interesting to evaluate obesity related parameters, such as hepatosteatosis and adipose tissue hypertrophy in further studies. As hypertension was only observed in rats fed the HFSSD, high-salt is likely to be the contributing factor to its generation, an observation previously demonstrated in both humans and animals [30]–[33]. Given that rats fed HFSD and HFSSD both developed hyperinsulinemia and dyslipidemia (FFA), it is likely that multifactorial factors including high-fat, high-sucrose and even high-salt may contribute to the disruption of the metabolic homeostasis, leading to metabolic syndrome (IR, dyslipidemia and hypertension), which in turn is thought to increase the risk of developing T2DM and cardiovascular diseases [34]–[39]. In order to see if a prediabetic phenotype can be induced by diet, we measured FPG and calculated the HOMA-IR index [27], [40]. As expected, a distinct rise in the level of FPG and significant increase in the HOMA-IR index were observed in rats fed either high energy diet type. Thus, it would be rational to conclude that IR or prediabetes (IFG) is beginning to occur in these rats although the alterations noted did not reach the hyperglycemia thresholds required to diagnose prediabetes in humans [3], [6]. It will be interesting to see whether the level of glycosylated hemoglobin A1C can be altered by diet in the future, as this factor has recently been suggested to be useful biomarker for screening undiagnosed diabetes and prediabetes [41]. In some genetic or acquired rodent models of T2DM, hyperinsulinemia, dyslipidemia and IFG/IGT are observed (for details see Table S3). In addition, BioBreeding/Worcester, ZUC-fa/fa and ZDF-obese rats [42]–[46], high-fat diet C57BL6/J mice [47]–[48], leptin-deficient ob/ob and leptin receptor-deficient db/db mice [49] also display these symptoms.

The timing of onset of each metabolic component induced by high fat/sugar or high fat/sugar/salt often differed with diet-type. Interestingly, the onset of hyperinsulinemia induced by the HFSD was on PDD 15, while the onset of the FFA rise occurred on PDD 20 in rats fed both the HFSD and HFSSD. The occurrence of hyperinsulinemia earlier than FFA dyslipidemia may contradict the existing hypothesis that dyslipidemia (with production of more FFA) may be the cause of IR [50]–[51]. Nonetheless, the HFSD-induced FPG rise and the HFSD/HFSSD-associated increase in the HOMA-IR index both occurred by PDD 25. Collectively, high energy diets resulted in hyperinsulinemia and dyslipidemia (FFA) that may gradually lead to IR and prediabetic condition (IFG or IGT). The longitudinal and dynamic changes in insulin sensitivity (IR), lipid metabolism (dyslipidemia) and FPG level (IFG) induced by the high energy diets are therefore likely to be a chronic process and serve as risk factors of developing T2DM. Moreover, it was found that high dietary sodium associated hypertension occurs earlier than IR, dyslipidemia and IFG/IGT. Thus, this hypertensive predisposition may worsen the metabolic and prediabetic injury to both microvessels and macrovessels [32], [52]–[54], deteriorating the pathogenesis of diabetic complications including neuropathy and cardiovascular pathology [12], [14]–[15], [19], [24], [34], [55].

### High energy diets-induced somatic sensory dysfunction

Here we show that the occurrence of painful polyneuropathy may reflect an early consequence of diet-induced metabolic syndrome (hypertension, hyperinsulinemia, and dyslipidemia) and prediabetes (IFG or IGT).

Relative to CD, both the HFSD and HFSSD could induce bilateral reduction in PWMT but with no accompanying changes in PWTL. These results suggest the occurrence of mechanical hypersensitivity (hyperalgesia and allodynia) but not thermal hypersensitivity in rats fed high energy/salt diets. However, rats fed HFSD showed greater (about 2 fold more) mechanical hypersensitivity than those fed HFSSD, suggesting that the high salt-induced hypertension, or other effects, might produce a protective anti-hyperalgesic phenotype on the HFSD-induced sensory dysfunction [56]. In the high energy diet rats it was noted that the onset of the bilateral mechanical hypersensitivity was about a month later than the other plasma chemical changes. The onset delay between metabolic syndrome/prediabetes and somatic sensory changes indicates that sensory painful neuropathy may occur after chronic exposure to hyperinsulinemia, dyslipidemia (FFA), and IFG/or IGT, as proposed in humans [12], [14]–[15], [19], [24], [34], [55]. Furthermore, the perceived sensory changes (chronic pain levels) may be partially blocked by the addition of high-salt within the diet [56].

Motor coordinating performance of rats with HFSD and HFSSD, measured by Rota Rod treadmill on the last day of the experiment (PPD 120) declined significantly when compared with rats fed CD. The impaired motor coordinating performance by high energy/salt diets could be attributable to damage to either peripheral motor fibers in the sciatic nerve, the somatic proprioceptive ascending projection fibers along the spinal dorsal column or the central motor system after chronic exposure to hyperinsulinemia, dyslipidemia (FFA), and IFG/or IGT [13], [16]–[18], [57]–[58]. These changes do not however confound mechanical pain-like sensitivity seen in the high energy diet animals, as these animals flinch more in response to a given stimulus, relative to those on control diets.

Painful peripheral neuropathy is a major type of neuropathic pain that has been recently redefined by NeuPSIG (Neuropathic Pain Special Interest Group of the International Association for the Study of Pain) as ‘pain arising as a direct consequence of a lesion or disease affecting the somatosensory system’ [59]–[61]. The prevalence of pain in patients with diabetes has been reported to be about 25% [17], [60]. In clinic, painful peripheral neuropathy in patients with T1DM has been shown to be effectively relievable or reversible by blood glucose control, whereas the same treatment is not effective in patients with T2DM [12], [34]. Therefore as an alternative therapy, anticonvulsants and antidepressants have been suggested for pain management in patients with T2DM [12], [62]–[65]. Due to these observations, and others, it has gradually been recognized that the disparity in therapies for pain management between T1DM and T2DM reflect differential mechanisms of painful peripheral neuropathy between the two conditions. Confusion on what each animal model of diabetes is actually measuring T1DM and/or T2DM has added to a lack of clarity on the basic mechanisms of each diabetic syndrome and the subsequent etiology of their consequences [42, 45, 48, 66–86, for details see Table S3].

### High energy diets-induced metabolic and prediabetic painful polyneuropathy

The primary finding of the current study is that high energy/salt diets can induce disruption or breakdown of myelin sheath and axonal degeneration in the LMF of both the peripheral and central branches of the dorsal root ganglion neurons. As damage to large-myelinated motor fibers in the sciatic nerves could not be excluded, motor neuropathy is also possible and indeed suggested by reduced motor activity of both high energy/salt diet animals relative to controls. In contrast, the SMF and the UMF in the sciatic nerves remained mostly intact in rats fed HFSD and HFSSD although axolemma thickening and intra-axonal plasmic lipofuscin depositions could be occasionally found in UMF, especially in rats fed HFSSD. Similarly, the damage to the LMF was more extensive in rats fed HFSSD than those fed HFSD, suggesting that besides high energy components of the diets, high dietary sodium and/or its resultant hypertension are probably other important factors contributing to the pathogenesis of the LMF neuropathies. Even though levels of LMF damage were highest in the HFSSD rats, the onset and level of peripheral neuropathy (measured as tactile but not thermal hypersensitivity) was lower in these animals, suggesting that a high-salt diet maybe in some way protective to levels of pain-like tactile allodynia experienced by the rats, but not to LMF damage. At first these results may seem contradictory, however patients with similar amounts of nerve damage can experience markedly different levels of chronic pain, and the mechanisms that create these disparities are poorly understood [87]. Therefore although high-salt diet clearly does not help myelin structure within the PNS or CNS, results within these studies suggest that it may reduce the levels of chronic pain-like input perceived by these animals [56]. One could envisage for example, that perturbations in tissue sodium ion/chloride levels could possibly reduce action potential generation within these nerves in addition to exacerbating neural myelin damage. Although further work will be required to determine such possible parallel mechanisms, results presented here suggest only a temporary reprieve from chronic pain onset by high salt intake and one in the face of worse nerve damage overall. These results offer the opportunity to determine in patients any protective link between high salt in-take on a high energy diet background, temporary or otherwise, with respect to chronic pain perception.

Here we show pre-diabetic associated myelin damage in the dorsal columns of the spinal cord, however it has recently been recognized that IR may also lead to myelin injury or white matter lesions in the brain [88]–[89]. Severe white matter lesions detected by diffuse tensor imaging were positively correlated with metabolic syndrome (triglycerides, SBP and diastolic BP, and FPG, and reduced high-density lipoprotein-cholesterol) [90]–[92]. High-fat diets could also result in IR-associated cognitive decline in animals [93]. In addition high dietary sodium-induced hypertension may cause vascular inflammation in the brainstem where respiratory and cardiovascular centers are localized [32], [53] consequently leading to, autonomic neuropathy and other dysfunctions frequently observed following diabetes [94]–[97].

## Conclusions

In the current study, early metabolic syndrome (hyperinsulinemia, dyslipidemia, and hypertension) and prediabetic conditions (IFG) could be induced by high energy (high-fat and high-sucrose) diets in rats which later developed painful polyneuropathy that was characterized by myelin breakdown and LMF loss in both peripheral and central branches of primary afferent neurons. However, SMF and UMF were far less damaged in the same rats. The phenomenon that the high energy diets only induce mechanical, but not thermal, pain hypersensitivity may reflect a selective damage to LMF, but not to the SMF and UMF. Moreover, dietary sodium (high-salt) deteriorates the neuropathic pathological process induced by high energy diets further, but paradoxically high salt consumption may improve, at least temporarily, chronic pain perception in these animals.

We have therefore established a strong link between high-energy/high-salt diet induced metabolic syndrome and prediabetes which results in relatively selective LMF damage in both the PNS and CNS that in turn can result in neuropathic pain. These results have a profound impact on patient welfare relative to diet choice, not just for T2DM onset, but also for its associated neuropathic symptoms.

## Supporting Information

Figure S1 **Grading classification of pathological changes in the myelinated nerve fibers of the sciatic nerves (A) and the spinal dorsal column (B) from the HFSD and the HFSSD groups of rats.** I, a slight pathological change including myelin lamina rarefaction, focal demyelination or vacuolization with the axon being less affected; II, moderate pathological changes including myelin lamina reticulation, focal demyelination, vacuolization and axonal changes including increased electron density, lipofuscin deposition, glycogen granules; III, more severe pathological changes including wide myelin breakdown and axonal degeneration than what were seen in II; IV, the most severe pathological changes including dramatic myelin damage or disruption that are accompanied by axonal degeneration and loss. HFSD, high-fat and high-sucrose diets; HFSSD, high-fat, high-sucrose and high-salt diets. Scale bar in AI-IV: 5 µm; Scale bar in BI-IV: 2 µm.
(TIF)

Table S1 **The compositions of the CD, HFSD and HFSSD diets.**
(DOC)

Table S2 **The mass or energy proportion of carbohydrate, protein and fat in the CD, HFSD and HFSSD diets.**
(DOC)

Table S3 **Animal models of experimental, genetic and spontaneously-acquired diabetic, pre-diabetic and/or metabolic states with or without peripheral neuropathy.**
(DOC)
