# Supplementary figures and images for: High Energy Diets-Induced Metabolic and Prediabetic Painful Polyneuropathy in Rats
Source: PLoS One. 2013 Feb 25;8(2):e57427. doi: 10.1371/journal.pone.0057427 (PMC3581455; doi:10.1371/journal.pone.0057427)

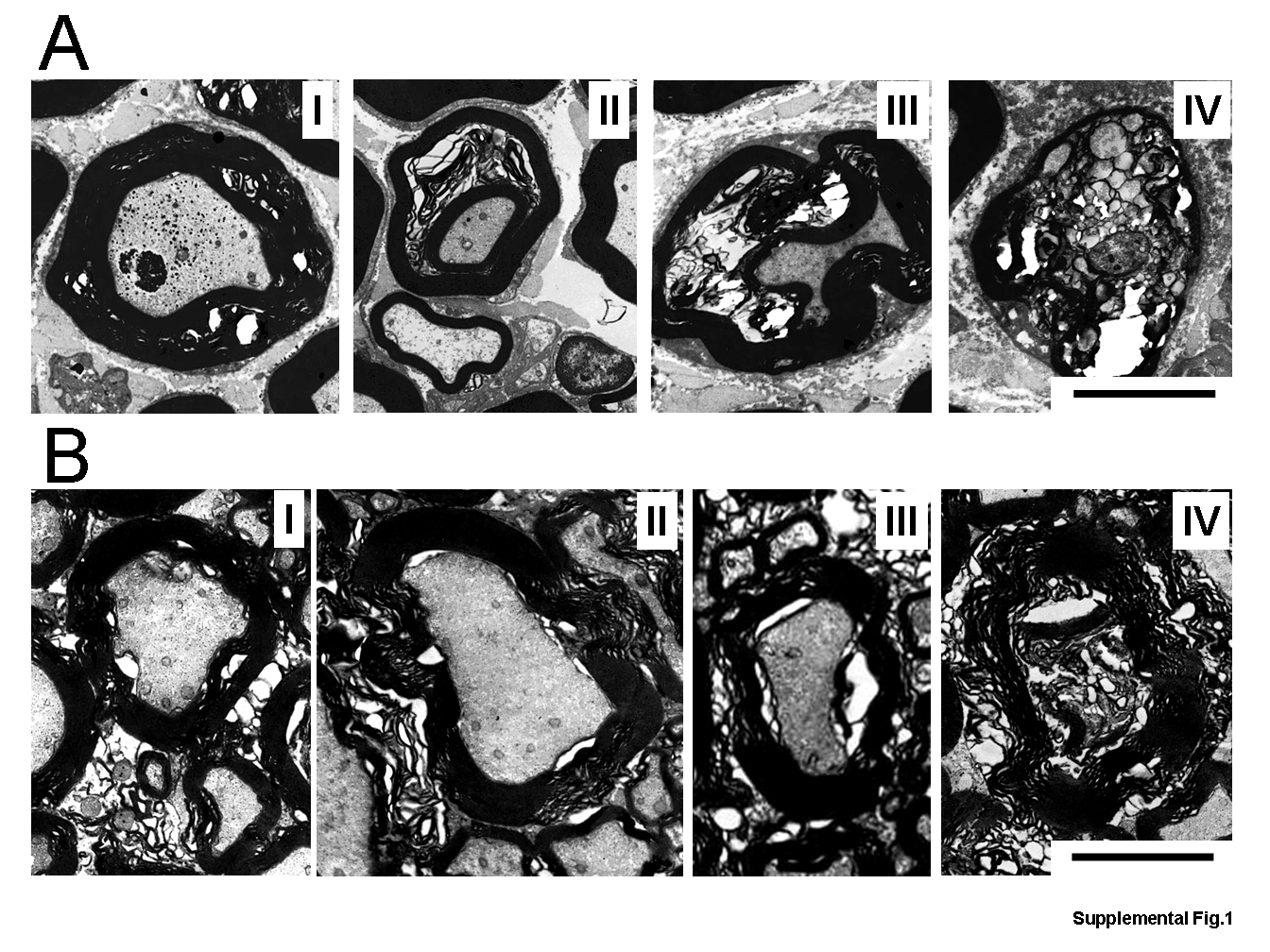

Supplement: Figure S1 — Grading classification of pathological changes in the myelinated nerve fibers of the sciatic nerves (A) and the spinal dorsal column (B) from the HFSD and the HFSSD groups of rats. I, a slight pathological change including myelin lamina rarefaction, focal demyelination or vacuolization with the axon being less affected; II, moderate pathological changes including myelin lamina reticulation, focal demyelination, vacuolization and axonal changes including increased electron density, lipofuscin deposition, glycogen granules; III, more severe pathological changes including wide myelin breakdown and axonal degeneration than what were seen in II; IV, the most severe pathological changes including dramatic myelin damage or disruption that are accompanied by axonal degeneration and loss. HFSD, high-fat and high-sucrose diets; HFSSD, high-fat, high-sucrose and high-salt diets. Scale bar in AI-IV: 5 µm; Scale bar in BI-IV: 2 µm. (TIF) [file pone.0057427.s001.tif]
