# Supplementary material for: High Energy Diets-Induced Metabolic and Prediabetic Painful Polyneuropathy in Rats
Source: PLoS One. 2013 Feb 25;8(2):e57427. doi: 10.1371/journal.pone.0057427 (PMC3581455; doi:10.1371/journal.pone.0057427)
Supplement: Table S1 — The compositions of the CD, HFSD and HFSSD diets. (DOC) [file pone.0057427.s002.doc]

Supplemental Table 1 The compositions of the CD, HFSD and HFSSD diets

| Ingredient | CD  (g/kg diet) | HFSD  (g/kg diet) | HFSSD  (g/kg diet) |
| --- | --- | --- | --- |
| *Sucrose (carbohydrate)* | *—* | *170* | *170* |
| Flour (carbohydrate) | 190 | 110 | 90 |
| Corn starch (carbohydrate) | 230 | 130 | 110 |
| Sorghum powder (carbohydrate) | 60 | 30 | 30 |
| Starch (carbohydrate) | 65 | 40 | 30 |
| Soybean powder (protein) | 150 | 80 | 70 |
| Fish flour (protein) | 100 | 50 | 50 |
| Bone meal (protein) | 10 | 5 | 5 |
| Bakers' yeast (protein) | 10 | 5 | 5 |
| *Yolk powder (35%protein +65%fat)* | *—* | *75* | *75* |
| *Lard (fat)* | *—* | *190* | *190* |
| Seed fat (fat) | 20 | 10 | 10 |
| Cod liver oil (fat) | 10 | 5 | 5 |
| Glycine (amino acid) | 35 | 20 | 20 |
| Methionine (amino acid) | 5 | 5 | 5 |
| CaCO_3_ (mineral matter) | 5 | 5 | 5 |
| *Dairy salt (mineral matter)* | 10 | 10 | 80 |
| Wheat bran (cellulose) | 100 | 60 | 50 |
| Total carbohydrate | 545 | 480 | 430 |
| Total protein (include amino acid) | 310 | 191.25 | 181.25 |
| Total fat | 30 | 253.75 | 253.75 |

Notes: CD, conventional diets; HFSD, high-fat and high-sucrose diets; HFSSD, high-fat, high-sucrose and high-salt diets; the italic stand for the additional components used for different composition of diets based upon that of conventional diets.

**Reference**

Pan, L., et al., 2000. Experimental syndrome of insulin resistance rat model. West China Med. J. 15, 421-422.
