# Supplementary material for: High Energy Diets-Induced Metabolic and Prediabetic Painful Polyneuropathy in Rats
Source: PLoS One. 2013 Feb 25;8(2):e57427. doi: 10.1371/journal.pone.0057427 (PMC3581455; doi:10.1371/journal.pone.0057427)
Supplement: Table S2 — The mass or energy proportion of carbohydrate, protein and fat in the CD, HFSD and HFSSD diets. (DOC) [file pone.0057427.s003.doc]

Supplemental Table 2 The mass or energy proportion of carbohydrate, protein and fat in the CD, HFSD and HFSSD diets

| Ingredients | CD | | HFSD | | HFSSD | |
| --- | --- | --- | --- | --- | --- | --- |
|  | Mass% | Energy% | Mass% | Energy% | Mass% | Energy% |
| Carbohydrate | 54.5% | 59.0% | 48.0% | 39.0% | 43.0% | 37.0% |
| Protein (include amino acids) | 31.0% | 34.0% | 19.1% | 15.0% | 18.1% | 15.0% |
| Fat | 3.0% | 7.0% | 25.3% | 46.0% | 25.3% | 48.0% |
| Total energy | 3.69 kcal/g | | 4.97 kcal/g | | 4.73 kcal/g | |

Notes: CD, conventional diet; HFSD, high-fat and high-sucrose diets; HFSSD, high-fat, high-sucrose and high-salt diets;
