# Supplementary material for: High Energy Diets-Induced Metabolic and Prediabetic Painful Polyneuropathy in Rats
Source: PLoS One. 2013 Feb 25;8(2):e57427. doi: 10.1371/journal.pone.0057427 (PMC3581455; doi:10.1371/journal.pone.0057427)
Supplement: Table S3 — Animal models of experimental, genetic and spontaneously-acquired diabetic, pre-diabetic and/or metabolic states with or without peripheral neuropathy. (DOC) [file pone.0057427.s004.doc]

Supplemental Table 3 Animal models of experimental, genetic and spontaneously-acquired diabetic, pre-diabetic and/or metabolic states with or without peripheral neuropathy

| Species | Experimental procedures | Variables of diabetic and pre-diabetic states | Neurological assessment | Electrophysiology | Histological assessment | References | | |
| --- | --- | --- | --- | --- | --- | --- | --- | --- |
| *Rodent* | | | | | | | | |
| Sprague-Dowley rat (3 m old, sex N/A) | Alloxan (120 mg/kg bw, i.p., observed till 6 m or 7-9 m old) vs. control | Type 1 DM  Hyperglycemia  Glycosuria  Body weight ↓ | Sensory: N/A  Motor: muscle atrophy | CV:  Sciatic ↓  CMAP amplitude ↓ (29%) | Sciatic:  Large MF loss  No axonal degeneration  Muscle fiber loss  Endplate loss | Ref 1 | | |
| Wistar rat (3 m old, male) | Streptozotocin (50 mg/kg, i.v., observed till 7 m old) vs. control (0.4 ml sodium citrate buffer, i.v.) | Type 1 DM  Hyperglycemia  Glycated hemoglobin ↑  Body weight ↓ | N/A | CV:  Tibial ↓ | Sural>Sciatic>Dorsal root:  MF atrophy with neurofilament loss:  Fiber size ↓  Fiber density ↑  Axon/Fiber size↓ | Ref 2 | | |
| Rat (250-600 g, male) | Streptozotocin (50-70 mg/kg, i.p., observed for 1 year) or alloxan (125-150 mg/kg, i.p., observed for 1 year)  vs. control (matched age) | Type 1 DM  Hyperglycemia  Glycosuria | No sensory and motor loss | CV:  Sciatic ↓ | Sural and tibial:  No change in MF  Number -  Size -  Myelin thickness -  Internodal length -  No change in UMF (vagus)  Schwann cell basal laminal thickness -  Microvessel - | Ref 3 | | |
| C57BL/6  (6 w old, male) | Streptozotocin (180 mg/kg, i.p., observed till 8 w old) vs. control without any treatment | Type 1 DM  Hyperglycemia  Body weight ↓ | Hypoalgesia  PWMT ↑  PWTL ↑ | CV: *In vitro* skin-nerve  Aβ unchanged  Aδ unchanged  C unchanged  AP response to vF:  RA Aβ ↓  SA Aβ unchanged  SA Aδ unchanged  C ↓  vFT:  RA Aβ ↓^n.s^.  SA Aβ unchanged  SA Aδ ↑^n.s^.  C ↑^n.s^.  hT of C: ↑ | Saphenous  Aβ fiber loss  Dramatic axon-myelin separation | Ref 4 | | |
| thy1-YFP (line thy1-YFP16) transgenic mice, 6 w old, male) | Streptozotocin  (200 mg/kg, i.p.) | Type 1 DM  Hyperglycemia (mmol/L)  1 m: 31.9±0.7  2 m: 31.4±0.5  3 m: 30.6±1.1  6 m: 27.4±1.9  Body weight ↓ | Hypoalgesia  PWTL ↑  PWMT^N/A^ | CV:  Sciatic ↓ | Fluorescent image:  Cutaneous YFP fiber ↓  Skin biopsy:  Cutaneous small fiber ↓  PGP9.5 ↓  CGRP ↓  SP ↓  P2X3 ↓ | Ref 5 | | |
| Sprague-Dawley rat (240-300 g, female) | High galactose feeding (40%, diet, observed for 2, 4, 24 m) | Type 1 DM  Hyperglycemia (2 m) | N/A | N/A | Reactive SC:  Lipid droplets  π granules of Reich  Glycogen granules  Subplasmalemmal caveolae  Cytoplasmic expansion, Capping  Degenerative SC:  Mit enlargement  Mit crista loss  Abaxonal and adaxonal disintegration  Proliferative SC:  Mitotic figures  Bungner’s bands  Onion-bulb  Remyelination | Ref 6 | | |
| BioBreeding/Worcester rat | BB/Wor rat develops diabetes spontaneously as a result of an autoimmune-mediated β-cell destruction (onset: 73±2 days of age till 10 m) vs. non-diabetic-prone BB rat | Type 1 DM  Hyperglycemia  Insulin ↓ (88%)  C-peptide ↓ (96%)  IGF-I ↓ (37%)  Body weight ↓ (28%) | Thermal hyperalgesia  PWTL ↓ (60%)  PWMT ^N/A^ | CV:  Tibial ↓ | DRG neuron loss (26%)  MF loss (30%)  UMF loss (56%)  SP neuron ↓ (45%)  CGRP neuron ↓ (48%)  Swollen Golgi apparatus  Active caspase-3 ↑  HSP70 ↓  NGF receptor ↓ | Refs 7-9 | | |
|  | eu-/ hypoglycaemic insulin-treated rats vs. control rats (4-6 m old) | Hypoglycaemia | N/A | N/A | Lateral plantar nerve  Large MF loss  Denervated SC ↑ | Ref 10 | | |
| BioBreeding/Worcester rat (male) | BB/Wor rat (onset: 71±3 days) vs. nondiabetic BB/Wor Rat | Type 1 DM:  Glucose ↑  Glycated Hb ↑  Insulin ↓ (88%)  C-peptide ↓  IGF-1 ↓  Body weight ↓ (24%) | Hyperalgesia:  PWTL ↓↑ | CV:  Hind-limb sensory nerve ↓ | Sural:  UMF loss (50%)  Denervated SC ↑  Collagen poket ↑  Dorsal root:  Insulin receptor ↓  Sciatic:  NGF, NT-3, SP, CGRP ↓ | Ref 11 | | |
| BioBreeding ZDR/Wor rat (male) | Type 2 DM BBZDR/Wor rat (onset: 76±4 days) vs. nondiabetic BB/Wor Rat | Type 2 DM  Glucose ↑  Glycated Hb ↑  Insulin ↑ (36%)  C-peptide ↑ (11%)  IGF-1 ↓  Body weight ↑ (17%) | Hyperalgesia:  PWTL ↓ | CV:  Hind-limb sensory nerve - | Sural:  UMF loss (9%)  Collagen poket ↑  Dorsal root:  Insulin receptor -  Sciatic:  NGF, NT-3, SP, CGRP - |  |  |  |
|  |  |  |  |  |  |  |  |  |
| Zucker diabetic fatty rat (5 w old, sex ^N/A^) | ZDF (onset: 8 w old and observed till 19 w old) vs. ZL | Type 2 DM  14 w old:  Glucose (mmol/L)↑  ZDF: 28.4±2.9  ZL: 6.0±0.1  19 w old:  Glucose (mmol/L) ↑↑  ZDF: 436.6±30  ZL: 394±6.5  Body weight ↓ | Thermal hyperalgesia  PWTL ↓  PWMT - | N/A | N/A | Ref 12 | | |
| Zucker diabetic fatty rat (6 w old, male) | ZDF/crl-lepr/fa (onset: 8 w)  vs. lean fa/+ (ZL) | Type 2 DM  8 w old:  Glucose (mmol/L) ↑  ZDF: 12.6±0.9  ZL: 4.5±0.2  16 w old:  Glucose (mmol/L) ↑↑  ZDF: 397±10  ZL: 447±8  Body weight ↓ | Mechanical allodynia:  PWMT ↓  PWTL - | CV (4 m):  Sciatic-tibial ↓ | Intradermal axonal loss  NF200 ↓  PGP9.5 ↓ | Ref 13 | | |
| Zucker diabetic fatty rat (6 w old, male) | ZUC-fa/fa vs. ZL (Crlj:ZUC-+/?)  ZDF/GmiCrl-fa/fa vs. ZL (ZDF/GmiCrl-+/?) | Obesity-associated IR (8-36 w old):  Obesity  Hyperinsulinemia  Glucose ↑  Body weight ↑    Type 2 DM (8-36 w)  Hyperinsulinemia  Glucose ↑↑  Body weight ↑↓ | TFL ↓ ↑  PWMT^RST^ –  TFL ↓ - ↑  PWMT^RST^ - ↓ | N/A | N/A | Ref 14 | | |
| Zucker diabetic fatty rat (5 w old, male) | ZF/Gmi-fa/fa  (6.5-9.5 w old)  ZDF/Gmi-fa/fa  ( 6.5-9.5 w old)  ZL+/+  (6.5-9.5 w old) | Pre-diabetes  Fasting glucose ↑  Random glucose –  Body weight ↑↑  Type 2 DM  Fasting glucose ↑  Random glucose ↑↑ (since 9 w old)  Body weight ↑  Control  Fasting glucose –  Random glucose –  Body weight ↑ | PWMT^RST^ ↓  PWMT^RST^ ↓↓  PWMT^RST^ - | N/A | N/A | Ref 15 | | |
| Zucker diabetic fatty rat (6 w old, male) | Zucker vs. ZDF-lean (+/?)  ZDF-obese (fa/fa) vs. ZDF-lean (+/?) | Hyperinsulinemia (8-32 w)  FFA ↑  Triglycerides ↑  Body weight ↑ (8-40 w)    Hyperglycemia (8-40 w)  FFA ↑  Triglycerides ↑  Body weight - | N/A | CV:  Motor ↓ (32-40 w)  Endoneurial blood flow ↓ (28-40 w)  CV:  Motor ↓ (24-40 w)  Endoneurial blood flow ↓ (12-40 w) | Sciatic:  Ach-mediated vascular relaxation ↓ (16 w)  CGRP-mediated vascular relaxation –  Oxidative stress:  Superoxide ↑  Peroxynitrite ↑  Sciatic:  Ach-mediated vascular relaxation ↓ (8-10 w)  CGRP-mediated vascular relaxation ↓ (28 w)  Oxidative stress:  Superoxide ↑  Peroxynitrite ↑ | Ref 16 | | |
|  | | | | | | | | |
| C57BL6/J mice (20-21 g, female) | High-fat diet  (fed for 16 w) vs. control with standard mouse chow | Pre-diabetic or Type 2 DM  Glucose ↑ (3.9%)  Insulin ↑ (68%)  FFA ↑ (98%)  Body weight ↑ (27%) | Hypoalgesia  PWTL ↑ (68%)  Hyperalgesia  PWMT ↓ (46%) | CV:  Sciatic ↓ | MF unchanged  IDNF: unchanged | Ref 17 | | |
| C57BL6/J mice (23-25 g, male) | High-fat diet  (fed for 16 w or 23 w) vs. control with normal chow | Pre-diabetic or obesity  Glucose ↑ (14.5%)  Body weight ↑ (40%) | Hypoalgesia  PWTL ↑ (59%)  TFL ↑ (86%)  RST ↑ 25%)  Tactile allodynia  vFT ↓ (38%) | CV:  Sciatic ↓  Motor ↓ (6%)  Sensory ↓ (14%) | Oxidative-nitrosative stress:  Lipoxygenase ↑  12(S)-HETE ↑  Nitrotyrosine ↑ | Ref 18 | | |
| C57BL6/J mice (3 w old, male) | High-fat diet  (fed for 34 w) vs. control with normal chow | IGT:  Hyperinsulinemia  Glycosylated Hb ↑  Dyslipidemia:  Hypertriglyceridemia  Cholestrol ↑  Oxidative-nitrosative stress:  HODE ↑  Dityrosine ↑  Nitrotyrosine ↑  Body weight ↑ | Hypoalgesia  PWTL ↑ | CV:  Sural ↓  Sciatic ↓ | Biopsy:  IDNF ↓  Sciatic:  HODE ↑  Dityrosine ↑  Nitrotyrosine ↑  DRG neuron oxidative stress  Caspase 3 ↑  TUNEL ↑ | Ref 19 | | |
| Leptin-deficient ob/ob mice and leptin receptor-deficient db/db mice (4 m old, both sex) | vs. WT-C57BL6 | Type 2 DM  Glucose [Fasting (Post-load)]:  ob/ob: 7.6±1.2 (10.9±1.4)  db/db: 16.3±3.7 (25.5±6.4)  WT: 4.9±0.9 (5.8±1.1)  Insulin:  ob/ob: 21.2±4.5  db/db: 38.7±6.1  WT: 7.2±0.5  FFA:  ob/ob: 2.23±0.9  db/db: 3.51±1.5  WT: 1.5±0.4  Body weight:  ob/ob: 56-59 g  db/db: 59-61 g  WT: 25-27 g | N/A | N/A | Myelin sheath thickness:  ob/ob ↓ (only for large MF)  db/db ↓ (all sizes)  Basement membrane of endoneural microvesels:  ob/ob ↑ (34%)  db/db ↑ (33%)  Oxidative stress:  ob/ob ↑  db/db ↑ | Ref 20 | | |
| Transgenic mice (Ins.D^d^1) with hypoinsulinemic diabetes | Male-specific β-cell loss by increased expression of a syngeneic MHC Class I protein in pancreas | Type 1 DM (2-7 m old)  Hyperglycemia | N/A | CV (4-7 m):  Sciatic and tibial ↓  C fiber amplitude ↓ | Tibial:  MF damage  UMF loss  Intraneural space ↑ | Ref 21 | | |
| *Feline* | | | | | | | | |
| Cat  (7-15 years old, both sex) | Spontaneously occurring condition | Pre-diabetes or Type 1 DM  Hyperglycemia  Fructosamine ↑ (31-63%)  Glycosylated Hb↑ (69%)  Insulin ^N/A^  Body weight ↑ (20%) | Allodynia  Neurological defect: plantigrade posture when standing and walking; avoiding jump  Muscle atrophy  Decreased tendon reflexes | CV:  Peroneal ↓ (51%)  CMAP amplitude ↓ (65%) | Peroneal biopsy:  MF loss and axonal dystrophy  density ↓ (50%)  diameter ↓ (30%)  Pathological changes:  Paranodal demyelination  Segmental demyelination  Wallerian degeneration  Capillary luminal size ↑  Basement membrane thickening | | | Refs 22-25 |
| *Canine* | | | | | | | | |
| Beagle dog (3-4 m old, male) | Injection of a mixture containing alloxan (40 mg/kg) and streptozotocin (35 mg/kg), i.v. | Type 1 DM  Hyperglycemia  HbA1c ↑  Body weight ↑ | N/A | N/A | MF loss  UMF loss  Microvessel basement membrane area ↑ | | Ref 26 | |
| Dogs (4-8 years old, both sex) | Spontaneously diabetic dogs (since 6 m old of age) vs. control (body weight- and sex- matched) | Type 1 DM  Hyperglycemia  HbA1c ↑ | N/A | CV:  Tibial ↓^ns^ | Perineurial changes:  Sheath thickness ↑  Lamellar number↑  Interlamellar space ↑  Basement membrane thickness ↑ | | Ref 27 | |

Abbreviations: AP, action potential; Ach, acetylcholine; CGRP, calcitonin gene-related peptide; CMAP, compound muscle action potential; CV, conduction velocity (m/s); DRG, dorsal root ganglion; FFA, free fatty acid; HETE, hydroxyeicosatetraenoic acid; HODE, hydroxyoctadecadienoic acid; HSP, hot shock protein; hT, heat threshold (˚C); IDNF, Intradermal nerve fiber; IGF, insulin growth factor; IGT, impaired glucose tolerance; i.p., intraperitoneal; IR, insulin resistance; i.v., intravenous; m, month; MF, myelinated fiber; Mit, mitochondrial; N/A, not available; NGF, nerve growth factor; ns, not significant; NT, neurotrophin; P2X3, purinergic recptor 2 X 3 subtype; PGP9.5, protein gene product 9.5; RA, rapidly adapting; RST, Randall-Selitto test; SA, slowly adapting; SC, Schwann cell; SP, substance P; TFL, tail flick latency; TUNEL, TdT-mediated dUTP-biotin nick-end labeling; Type 1 DM, type 1 diabetes mellitus; Type 2 DM, type 2 diabetes mellitus; UMF, unmyelinated fiber; vFT, von Frey threshold; w, week; YFP, yellow fluorescent protein; ZDF, Zucker diabetic fatty; ZDR, Zucker diabetic rat; ZL, Zucker lean; ZUC, Zucker. ↑, increase; ↓, decrease, −, no change.

**References**

1. Hildebrand J, Joffroy A, Graff G, Coers C (1968) Neuromuscular changes with alloxan hyperglycemia. Electrophysiological, biochemical, and histological study in rats. Arch Neurol 18 (6):633-641
2. Yagihashi S, Kamijo M, Watanabe K (1990) Reduced myelinated fiber size correlates with loss of axonal neurofilaments in peripheral nerve of chronically streptozotocin diabetic rats. Am J Pathol 136 (6):1365-1373
3. Sharma AK, Thomas PK (1974) Peripheral nerve structure and function in experimental diabetes. J Neurol Sci 23 (1):1-15
4. Lennertz RC, Medler KA, Bain JL, Wright DE, Stucky CL (2011) Impaired sensory nerve function and axon morphology in mice with diabetic neuropathy. J Neurophysiol 106 (2):905-914
5. Chen YS, Chung SS, Chung SK (2005) Noninvasive monitoring of diabetes-induced cutaneous nerve fiber loss and hypoalgesia in thy1-YFP transgenic mice. Diabetes 54 (11):3112-3118
6. Kalichman MW, Powell HC, Mizisin AP (1998) Reactive, degenerative, and proliferative Schwann cell responses in experimental galactose and human diabetic neuropathy. Acta Neuropathol 95 (1):47-56
7. Sima AA (1980) Peripheral neuropathy in the spontaneously diabetic BB-Wistar-rat. An ultrastructural study. Acta Neuropathol 51 (3):223-227
8. Sima AA, Garcia-Salinas R, Basu PK (1983) The BB Wistar rat: an experimental model for the study of diabetic retinopathy. Metabolism 32 (7 Suppl 1):136-140
9. Kamiya H, Zhang W, Sima AA (2006) Degeneration of the Golgi and neuronal loss in dorsal root ganglia in diabetic BioBreeding/Worcester rats. Diabetologia 49 (11):2763-2774
10. Mohseni S, Hildebrand C (1998) Hypoglycaemic neuropathy in BB/Wor rats treated with insulin implants: electron microscopic observations. Acta Neuropathol 96 (2):151-156
11. Kamiya H, Murakawa Y, Zhang W, Sima AA (2005) Unmyelinated fiber sensory neuropathy differs in type 1 and type 2 diabetes. Diabetes Metab Res Rev 21 (5):448-458
12. Piercy V, Banner SE, Bhattacharyya A, Parsons AA, Sanger GJ, Smith SA, Bingham S (1999) Thermal, but not mechanical, nociceptive behavior is altered in the Zucker Diabetic Fatty rat and is independent of glycemic status. J Diabetes Complications 13 (3):163-169
13. Brussee V, Guo G, Dong Y, Cheng C, Martinez JA, Smith D, Glazner GW, Fernyhough P, Zochodne DW (2008) Distal degenerative sensory neuropathy in a long-term type 2 diabetes rat model. Diabetes 57 (6):1664-1673
14. Sugimoto K, Rashid IB, Kojima K, Shoji M, Tanabe J, Tamasawa N, Suda T, Yasujima M (2008) Time course of pain sensation in rat models of insulin resistance, type 2 diabetes, and exogenous hyperinsulinaemia. Diabetes Metab Res Rev 24 (8):642-650
15. Romanovsky D, Walker JC, Dobretsov M (2008) Pressure pain precedes development of type 2 disease in Zucker rat model of diabetes. Neurosci Lett 445 (3):220-223
16. Oltman CL, Coppey LJ, Gellett JS, Davidson EP, Lund DD, Yorek MA (2005) Progression of vascular and neural dysfunction in sciatic nerves of Zucker diabetic fatty and Zucker rats. Am J Physiol Endocrinol Metab 289 (1):E113-122
17. Obrosova IG, Ilnytska O, Lyzogubov VV, Pavlov IA, Mashtalir N, Nadler JL, Drel VR (2007) High-fat diet induced neuropathy of pre-diabetes and obesity: effects of "healthy" diet and aldose reductase inhibition. Diabetes 56 (10):2598-2608
18. Watcho P, Stavniichuk R, Ribnicky DM, Raskin I, Obrosova IG (2010) High-fat diet-induced neuropathy of prediabetes and obesity: effect of PMI-5011, an ethanolic extract of Artemisia dracunculus L. Mediators Inflamm 2010:268547
19. Vincent AM, Hayes JM, McLean LL, Vivekanandan-Giri A, Pennathur S, Feldman EL (2009) Dyslipidemia-induced neuropathy in mice: the role of oxLDL/LOX-1. Diabetes 58 (10):2376-2385
20. Nowicki M, Kosacka J, Serke H, Bluher M, Spanel-Borowski K (2012) Altered sciatic nerve fiber morphology and endoneural microvessels in mouse models relevant for obesity, peripheral diabetic polyneuropathy, and the metabolic syndrome. J Neurosci Res 90 (1):122-131
21. Elias KA, Cronin MJ, Stewart TA, Carlsen RC (1998) Peripheral neuropathy in transgenic diabetic mice: restoration of C-fiber function with human recombinant nerve growth factor. Diabetes 47 (10):1637-1642
22. Mizisin AP, Shelton GD, Wagner S, Rusbridge C, Powell HC (1998) Myelin splitting, Schwann cell injury and demyelination in feline diabetic neuropathy. Acta Neuropathol 95 (2):171-174
23. Mizisin AP, Shelton GD, Burgers ML, Powell HC, Cuddon PA (2002) Neurological complications associated with spontaneously occurring feline diabetes mellitus. J Neuropathol Exp Neurol 61 (10):872-884
24. Mizisin AP, Nelson RW, Sturges BK, Vernau KM, Lecouteur RA, Williams DC, Burgers ML, Shelton GD (2007) Comparable myelinated nerve pathology in feline and human diabetes mellitus. Acta Neuropathol 113 (4):431-442
25. Estrella JS, Nelson RN, Sturges BK, Vernau KM, Williams DC, LeCouteur RA, Shelton GD, Mizisin AP (2008) Endoneurial microvascular pathology in feline diabetic neuropathy. Microvascular research 75 (3):403-410
26. Walker D, Siddique I, Anderson H, Gardiner TA, Archer DB, Boulton AJM, Malik RA (2001) Nerve pathology in the type 1 diabetic dog: effects of treatment with sulindac. J Peripher Nerv Syst 6 (4):219-226
27. Ghani M, Malik RA, Walker D, Sharma AK, Lowrie CT, Schall WD, Boulton AJ (1999) Perineurial abnormalities in the spontaneously diabetic dog. Acta Neuropathol 97 (1):98-102
